# Single-cell sequencing reveals the immune microenvironment associated with gastric cancer

**DOI:** 10.1016/j.gendis.2024.101218

**Published:** 2024-01-26

**Authors:** Guoquan Huang, Cheng Yuan, Chao Zhang, Fuyu Yang, Yong Tan, Defei Chen, Hui Li, Kun Qian

**Affiliations:** a Hubei Selenium and Human Health Institute, The Central Hospital of Enshi Tujia and Miao Autonomous Prefecture, Enshi, Hubei 445000, China; b Hubei Provincial Key Lab of Selenium Resources and Bioapplications, Enshi, Hubei 445000, China; c Department of Gastrointestinal Surgery, Central Hospital of Enshi Tujia and Miao Autonomous Prefecture, Enshi, Hubei 445000, China; d Department of Oncology, Yichang Central People's Hospital and The First College of Clinical Medical Science, China Three Gorges University Yichang, Hubei 443000, China; e General Surgery Department of Xuan'en County People's Hospital, Enshi, Hubei 445003, China; f Department of Gastrointestinal Surgery, The First Affiliated Hospital of Chongqing Medical University, Chongqing 400016, China

Gastric cancer has a high incidence worldwide; the incidence of gastric cancer ranks fifth among all malignant tumors, and the mortality rate ranks fourth.^1^ The immune microenvironment plays an important role in the occurrence, progression, and metastasis of gastric cancer.^2^ Immunotherapy has been recognized as the most effective treatment for a variety of human cancers. Among immunotherapy strategies, the blockade of immune checkpoints is becoming the most common cutting-edge cancer immunotherapy method, and also the most effective method.^3^ Given the high incidence and mortality of gastric cancer, identifying new immune checkpoints in the process of gastric cancer formation is urgently needed, as it provides a new theoretical basis for the diagnosis and treatment of gastric cancer. Thus, we conducted single-cell sequencing analysis of three pairs of primary gastric cancer and adjacent normal tissues to search for genes related to the immune escape of gastric cancer cells. The detailed case data are shown in Table S1. Our results revealed that the VSIR gene plays an important role in the development of gastric cancer. However, the mechanism by which the VSIR gene helps gastric cancer cells achieve immune escape during gastric cancer formation is still unclear, and further research is needed.

We utilized the RunUMAP and RunTSNE functions in Seurat_4.0.4 for dimensionality reduction analysis of our single-cell sequencing data, directly extracted macrophage subsets, and subsequently analyzed macrophage subsets in detail to explore their role in the tumor microenvironment of gastric cancer and the formation of gastric cancer (Fig. 1A, B). First, myeloid cells were sorted through the LYZ and MNDA genes, which are myeloid cell marker genes (Fig. 1C). Then, we selected monocytes expressing VCAN, FCN1, CD14, and FCGR3A as gene markers (Fig. 1D); macrophages expressing C1QA, MRC1, MARCO, CD68, CD163, and APOE as markers (Fig. 1E); and neutrophils expressing CSF3R, CXCR2, and FCGR3B as marker genes. Dendric cells were divided into cDC1s and cDC2s, which were sorted by XCR1, CLEC9A, and THBD and CD1C, CD1E, and FCER1A as marker genes. Mast cells were screened using TPSAB1, TPSB2, and CPA3 as genetic markers. Further statistical analysis was performed to determine the number of genes and marker genes for each cell type (Fig. 1F), and we found many differentially expressed genes (DEGs) related to the formation of gastric cancer through differential gene analysis. The DEGs in monocytes are shown in the volcano map and Table S1. For example, CD44, FTH1, TIMP1, SOD2, VSIR, and SDC2 were identified (Fig. 1G and Table S2). We subsequently found numerous DEGs related to macrophages related to the development of gastric cancer, such as CST3, MS4A6A, CTSS, MPEG1, VSIR, and SDC2 (Fig. 1H and Table S3). Furthermore, we analyzed the genes related to the differential expression of neutrophils during the development of gastric cancer, for instance, CXCR2, CMTM2, FCGR3B, CXCR1, SDC2, and FABP5 (Fig. 1I and Table S4). Since inflammation plays an important role in the formation of gastric cancer, we further analyzed macrophages and plasma cells and compared the DEGs between them to determine which genes were significant. The results showed that CD79A, DERL3, TNFRSF17, FKBP11, VSIR, SDC2, and other genes play vital regulatory roles in the formation of gastric cancer (Fig. 1J and Table S5). The above results revealed that the VSIR gene plays an important role in immune regulation during the development of gastric cancer. However, the mechanism through which it promotes the immune escape of gastric cancer cells during the formation of gastric cancer has not yet been studied. Therefore, we need to explore the immunoregulatory mechanism of the VSIR gene in the formation of gastric cancer in detail. This study provides a new target and theoretical basis for clinical immunotherapy in gastric cancer. Recent studies have shown that macrophages play vital roles in the formation and progression of gastric cancer. These can be divided into “classic” (M1) and “alternative” (M2) activation. M1 macrophages have anti-tumor effects and can kill pathogens and tumor cells; M2 macrophages can promote tumor formation. When they are activated, they can activate metabolic pathways that inhibit the adaptive immune response.^4^ Therefore, we further sorted the sequencing data of M1 (CD68, IL-1B, IL-6, and TNF-α) and M2 (MRC1, CD163, TGFB1, IL-10, and FN1) macrophages (Fig. 1K). Moreover, we analyzed the DEGs between M1-type and M2-type macrophages (Fig. 1L and Table S6). Furthermore, through precise sorting of gastric cancer cells, single-cell sequencing analysis revealed that the expression level of the VSIR gene in gastric cancer tissues was significantly greater than that in normal tissues (Fig. 1M). The expression levels of the VSIR gene in different types of cells were analyzed, and the results showed that the expression levels of the VSIR gene in macrophages, monocytes, and T-cells were abnormally elevated (Fig. 1N). The sequencing results suggest that the VSIR gene plays an important role in the development of gastric cancer, but the mechanism of action of this gene has rarely been studied. In the process of gastric cancer development, the underlying mechanisms enable gastric cancer cells to escape immune surveillance, so that they can constantly proliferate and eventually develop gastric cancer. During the data analysis, we found many significant cell-type interactions and cell-to-cell interactions. These cells include macrophages, T-cells, stromal cells, stem cells, epithelial cells, and master cells. Furthermore, bioinformatics analysis revealed numerous receptor-ligand genes associated with communication between cell types. We found, strikingly, that VSIR is involved in cell communication in both macrophages and T cells (Fig. 1O) and that VSIR can play an important role in the formation of ligands for CCL4L2 (Fig. 1P). We will conduct future experiments to explore these important findings in detail and determine the underlying molecular and biological mechanisms involved. In summary, our single-cell sequencing data analysis showed that the immune microenvironment plays an important role in the development of gastric cancer. Through single-cell sequencing analysis, we identified several new marker genes involved in the immune microenvironment and gastric cancer. The results showed that the VSIR gene may constitute a new immune detection point and immunotherapy target for gastric cancer. Moreover, through analysis, we found for the first time that the ligand of the VSIR gene is CCL4L2, but its mechanism of action is not clear at present. In subsequent experiments, we will further elucidate the mechanism by which the VSIR gene enables gastric cancer cells to generate immune escape and promote the formation and metastasis of gastric cancer through CCL4L2.

**Figure 1:**
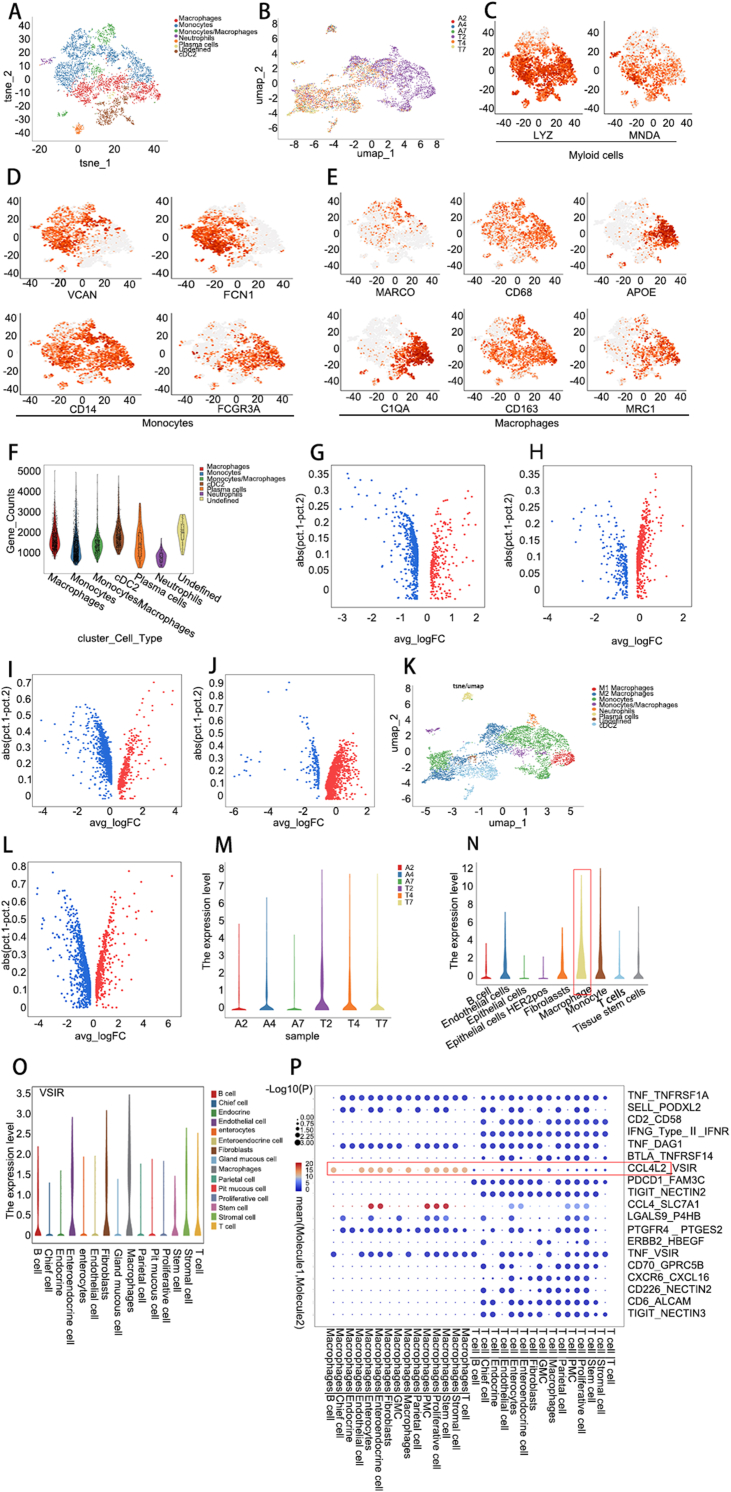
Single-cell sequencing reveals the immune microenvironment associated with gastric cancer. **(A)***t*-Distributed stochastic neighbor embedding (*t*-SNE) atlas of seven immune microenvironment-related cell subsets. **(B)** U-Map of scatterplots of annotated immune-associated cell subpopulations in three gastric cancer tissues and adjacent normal tissues. **(C)** Myeloid cells were sorted using the LYZ and MNDA genes. **(D)** Monocytes were sorted using the VCAN, FCN1, CD14, and FCGR3A genes. **(E)** Macrophages were sorted using the C1QA, MRC1, MARCO, CD68, CD163, and APOE genes. **(F)** The violin diagram showing the number of genes and marker genes for each cell type. **(G)** The volcano map showing the genes differentially expressed in monocytes. **(H)** The volcano map showing the genes differentially expressed in macrophages. **(I)** The volcano map showing the genes differentially expressed in neutrophils. **(J)** The volcano map showing the genes differentially expressed between the macrophage group and the plasma cell group. **(K)** The U-Map showing the M1 (CD68, IL-1B, IL-6, and TNF) and M2 (MRC1, CD163, TGFB1, IL-10, and FN1) macrophage subgroups. **(L)** The volcano map showing the genes differentially expressed between M1-type and M2-type macrophages. **(M)** The violin diagram showing the expression of the VSIR gene in three pairs of gastric cancer tissues and adjacent normal tissues. **(N)** The violin diagram showing the expression of the VSIR gene in different cell types. **(O)** The genes involved in cell communication in both macrophages and T cells. **(P)** The bubble maps showing the differences in ligand and receptor expression between M1-type and M2-type macrophages.

## Author contributions

Guoquan Huang, Cheng Yuan, Hui Li, and Kun Qian: research design. Guoquan Huang, Chao Zhang, and Cheng Yuan: data analysis. Guoquan Huang: experiments, manuscript writing, and figure drawing. Chao Zhang, Fuyu Yang, Yong Tan, and Defei Chen: specimen and clinical data collection and statistics. Guoquan Huang, Cheng Yuan, and Chao Zhang contributed equally to this work.

## Conflict of interests

All authors declared no conflict of interests.

## Funding

This study was funded by the Major Health Project of Science and Technology Department of Hubei Province, China (No. 2022BCE024) and Chongqing Natural Science Foundation Project (China) (No. cstc2021jcyj-msxmX0286). This work was also supported by grants from the National Natural Science Foundation of China (No. 82100763), Hubei Provincial Natural Science Foundation of China (No. 2021CFB090), the Fundamental Research Funds for the Central University (China) (No. 2042021kf0150), Zhongnan Hospital of Wuhan University Science, Technology and Innovation Seed Fund (China) (No. cxpy2020027), Excellent Doctor (Post), Zhongnan Hospital of Wuhan University (No. ZNYB2020009), and Wuhan Yingcai (China) (No. WHYCXQN2021004).
