# Supplementary material for: Single-cell sequencing reveals the immune microenvironment associated with gastric cancer
Source: Genes Dis. 2024 Jan 26;12(1):101218. doi: 10.1016/j.gendis.2024.101218 (PMC11466575; doi:10.1016/j.gendis.2024.101218)
Supplement: Multimedia component 7 [file mmc7.docx]

1. **Methods and Materials**

**2.1 Preparation, storage, transport of fresh samples and their dissolution into single cells**

The three pairs of gastric cancer samples in this study were obtained from the Department of Gastrointestinal Surgery, The First Affiliated Hospital of Chongqing Medical University. All patients provided written informed consent, and the study protocol was approved by the Ethics Review Committee of the First Affiliated Hospital of Chongqing Medical University. None of the patients received neoadjuvant chemotherapy before surgery. The normal tissue adjacent to the cancer was taken from more than 6.0 cm away from the border of the cancer tissue. The patient's clinical case data are detailed in Supplementary Table 1. The tissue sample, including cancer tissue and adjacent normal tissue (approximately 120 mg), was obtained, and the fresh samples were washed with phosphate-buffered saline (PBS) and preservation solution within the first five minutes. The specimen was then placed in a preservation solution (Singleron Bio Com, Nanjing, China) and transported to the laboratory, maintaining the cold chain, for dissolution into single cells. Fresh tissue was transferred to Petri dishes containing HBSS buffer with tweezers, washed 3 times to remove excess buffer and transferred to 1.5 mL enzyme-free EP tubes for accurate weighing. Only specimens meeting the following three conditions were used for experiments: separation time of less than 72 h, storage temperature 2-8 ℃, and sample mass of more than 100 mg. A small amount of Tissue Dissociation Mix was added to the tissue, and the tissue was minced and transferred to a new 15 mL centrifuge tube with 2.5 ml of Tissue Dissociation Mix. Next, enzymatic digestion was carried out in a constant temperature shaker (37 °C, 180 rpm) for 15 min, and 10 µl of the suspension was taken for trypan blue staining. The samples were considered fully mixed when cells were observed under a microscope without agglomeration. Finally, cell filtration was performed, and the cell concentration and viability were calculated using a fluorometer. When the cell viability was more than 85%, the total number of cells was more than 20,000, and the proportion of impurities or red blood cells was less than 20%, the sample was considered qualified for further analysis. Single-cell sequencing was then performed.

**2.2 Single-cell sample preparation, sequencing and bioinformatic analysis**

Samples used for single-cell sequencing were prepared according to the protocol of GEXSCOPE® Single-Cell Transcriptional Organism Library Kit (Singleron Bio Com, Nanjing, China), and the procedures included single-cell capture, cell lysis, molecular labeling, and cellular mRNA capture. cDNA was subjected to fragmentation, adapter ligation and subsequent steps to construct a sequencing library suitable for the sequencing platform. Then, single-cell sequencing was performed according to the protocol, and individualized bioinformatics analysis was carried out.

**2.3 Sample quality control and sequencing data processing**

The samples in this study were stringently evaluated according to the sample preparation and testing standard of cell suspension (Singleron Bio Com, Nanjing, China), as follows: A: a sample well-qualified for single-cell sequencing and is recommended for direct establishment of the library (cell number > 20000, cell activity > 85%); B: an equivocal sample carrying considerable risk for subsequent experiments (cell number > 20000, cell activity > 70%); C: a sample not qualified for single-cell sequencing, and subsequent experiments are not recommended (cell number < 20000, cell activity < 70%, or impurity:red blood cell ratio > 90%).

cDNA quality control standards: The test results and analysis are based on the company's (Singleron Bio Com, Nanjing, China) cDNA sample quality standard as follows: A. qualified sample: 30-100 ng, peak of quality inspection is between 900 bp-2000 bp, and 1000 bp > 15% for 5000 bp and < 40% for fragments below 300 bp; these samples are recommended for use to directly build the library; B: Equivocal samples: 900 bp-2000 bp quality inspection peak, not meeting any rating conditions of "qualified/unqualified;” C. unqualified: < 30 ng, quality inspection peak < 500 bp; these samples are not recommended for use to conduct the library building experiment,

Library quality control standards: The test results and analysis are based on the company's (Singleron Bio Com, Nanjing, China) library sample testing standard. The test results of this experiment are classified as follows: A. qualified: > 100 ng, the peak is between 300 bp-700 bp, and the ratio is 900 bp-5000 bp < 10%; the sample is recommended for use in experiments; B. equivocal: between 50 ng-100 ng, peak between 300 bp-700 bp, 900 bp-5000 bp accounting for 10% to 20%; C. unqualified: < 50 ng, main peak <300 bp, 300 bp-700 bp ratio < 30%; these samples are not recommended for use.

Data filtering and quality control: According to the expression matrix, the number of genes (nFeacture_RNA), the number of UMIs (nCount_RNA), the proportion of mitochondrial RNA, and the proportion of ribosomal RNA in each cell were counted. At the same time, abnormal cells were filtered according to the QC distribution of all cells: > filtered cells with less than 200 genes; > filtered cells with more than 6500 genes; > filtered cells with UMI number less than 500; > filtered cells with more than 50% mitochondrial RNA; > the proportion of filtered ribosomal RNA greater than 100% cells.

**2.4 Data dimensionality reduction,** **cell cluster analysis and cell annotation**

Cell cluster analysis: Based on the filtered cell expression matrix, the amount of gene expression in each cell was normalized (divided by the total expression, multiplied by 10000, and then log_10_) using the method in Seurat [1]. The data were then scaled, unnecessary sources of variation were removed [2], and dimensionality reduction and clustering were performed on the data [3]. We also performed multi-sample combined cell clustering analysis on gastric cancer tissues and adjacent normal tissues. By default, we used Seurat_V3 to perform combined cluster analysis on multicell samples. Furthermore, we mapped the sample information to a scatter plot in order to check the distribution of samples in subsets and to find the specific cell subsets of samples.

Cell Type Annotation: Annotation of cell types using single-R software, combined with marker gene subsets, helped us to identify the cell types represented by each subset. The software takes the existing purified cell type as a reference and calculates the correlation score between each cell and the reference to determine the cell type. A heatmap was used to show the scores of each subpopulation of cells against the reference cell type. We used this method to annotate gastric cancer tissues and adjacent normal tissues with multiple sets of cell types. Combining subsets of marker genes helped us to identify the cell types represented by each subset. The scatter plot shows cell types annotated by single-R software .

**2.5 Data quantification and statistical analysis**

**2.5.1 Differentially expressed gene analysis**

Different cell types or different samples were specified for differential gene analysis utilizing the ‘FindMarkers’ function, which conducts differential gene expression for two annotated cell groups using the nonparametric Wilcox rank sum test, and all cells except the control group, the default group and the treatment group were compared and analyzed. The marker genes were defined as at least twofold upregulated or downregulated compared to control cell expression levels.

**2.5.2 Enrichment analysis**

We conducted gene set and pathway enrichment analysis through Kyoto Encyclopedia of Genes and Genomes (KEGG) pathways and Gene Ontology (GO) terms for the focus gene list through the tool Enrichr [4].

**2.5.3 Exploring high-risk genes of gastric cancer**

The network relationship between disease phenotypes and genes was constructed according to CIPHER [5], and the driving genes of gastric cancer were inferred. In this analysis, we constructed a single-cell network: A node represents each cell type. The thickness of the borders represents Pearson’s correlation coefficient between the centroids of the cluster centroids between the two cell types (calculated by R's computer centroid function) for any pair of cell types.

**2.5.4 Trajectory analysis**

Pseudotime sequencing was performed on the selected cells to construct the pseudotime series locus, and the genes significantly associated with the pseudotime changes were found [6, 7]. For example, we performed a locus analysis of epithelial monocytes and macrophages, as shown in the figure. Subsequent studies will be conducted according to the data analysis.

**2.5.5 Tumor cell prediction**

We used copyKAT to predict solid tumor cells, generally without reference selection. When the number of cells exceeds 20,000, the program automatically screens cells randomly, screens 500 cells in each cluster, and then the tumor size is determined based on the cluster [8].

**2.5.6 Analysis of intercellular communication**

According to the cell ligand theory, a ligand acts on its receptor and thereby on the recipient cell, causing biological changes in the recipient cell. We used CellPhoneDB for cellular-interaction analysis to evaluate the interaction and communication between gastric cancer cells [9, 10].

**2.5.7 Differential analysis of cell heterogeneity**

Cell heterogeneity is a common biological phenomenon, and the characteristics of cells between normal cells and tumor tissues comprises a spectrum. During tumor development, the governing tumor cells may compose only a small part of the whole tumor tissue, and heterogeneity exists in the DNA, RNA and proteins of the cells. Cell heterogeneity analysis of these single-cell sequencing data was performed by inferCNV of the Trinity CTAT Project. <https://github.com/broadinstitute/inferCNV>.

**2.6 Statistical analysis**

In this study, we utilized SPSS software (version 26.0, IBM, USA) and GraphPad Prism (version 8.0, USA) to analyze all data. Analysis of single-cell sequencing data was completed through a single-cell data analysis platform. The results were determined to be statistically significant when the *p* value < 0.05.

1. Butler A, Hoffman P, Smibert P, Papalexi E, Satija R: **Integrating single-cell transcriptomic data across different conditions, technologies, and species**. *Nat Biotechnol* 2018, **36**(5):411-420.

2. Buettner F, Natarajan KN, Casale FP, Proserpio V, Scialdone A, Theis FJ, Teichmann SA, Marioni JC, Stegle O: **Computational analysis of cell-to-cell heterogeneity in single-cell RNA-sequencing data reveals hidden subpopulations of cells**. *Nat Biotechnol* 2015, **33**(2):155-160.

3. Xu C, Su Z: **Identification of cell types from single-cell transcriptomes using a novel clustering method**. *Bioinformatics* 2015, **31**(12):1974-1980.

4. Kuleshov MV, Jones MR, Rouillard AD, Fernandez NF, Duan Q, Wang Z, Koplev S, Jenkins SL, Jagodnik KM, Lachmann A *et al*: **Enrichr: a comprehensive gene set enrichment analysis web server 2016 update**. *Nucleic Acids Res* 2016, **44**(W1):W90-97.

5. Zhang P, Yang M, Zhang Y, Xiao S, Lai X, Tan A, Du S, Li S: **Dissecting the Single-Cell Transcriptome Network Underlying Gastric Premalignant Lesions and Early Gastric Cancer**. *Cell Rep* 2019, **27**(6):1934-1947 e1935.

6. Qiu X, Hill A, Packer J, Lin D, Ma YA, Trapnell C: **Single-cell mRNA quantification and differential analysis with Census**. *Nat Methods* 2017, **14**(3):309-315.

7. Trapnell C, Cacchiarelli D, Grimsby J, Pokharel P, Li S, Morse M, Lennon NJ, Livak KJ, Mikkelsen TS, Rinn JL: **The dynamics and regulators of cell fate decisions are revealed by pseudotemporal ordering of single cells**. *Nat Biotechnol* 2014, **32**(4):381-386.

8. Gao R, Bai S, Henderson YC, Lin Y, Schalck A, Yan Y, Kumar T, Hu M, Sei E, Davis A *et al*: **Delineating copy number and clonal substructure in human tumors from single-cell transcriptomes**. *Nat Biotechnol* 2021, **39**(5):599-608.

9. Efremova M, Vento-Tormo M, Teichmann SA, Vento-Tormo R: **CellPhoneDB: inferring cell-cell communication from combined expression of multi-subunit ligand-receptor complexes**. *Nat Protoc* 2020, **15**(4):1484-1506.

10. Vento-Tormo R, Efremova M, Botting RA, Turco MY, Vento-Tormo M, Meyer KB, Park JE, Stephenson E, Polanski K, Goncalves A *et al*: **Single-cell reconstruction of the early maternal-fetal interface in humans**. *Nature* 2018, **563**(7731):347-353.
